# Live imaging of apoptotic signaling flow using tunable combinatorial FRET-based bioprobes for cell population analysis of caspase cascades

**DOI:** 10.1038/s41598-022-25286-z

**Published:** 2022-12-07

**Authors:** Miho Suzuki, Yutaka Shindo, Ryu Yamanaka, Kotaro Oka

**Affiliations:** 1 Department of Applied Chemistry, Graduate School of Science and Engineering, Saitama University, Saitama, 338-8570 Japan; 2 Department of Bioscience and Informatics, Faculty of Science and Technology, Keio University, Kanagawa, 223-0061 Japan; 3 Faculty of Pharmaceutical Sciences, Sanyo-Onoda City University, Yamaguchi, 756-0884 Japan; 4 Graduate Institute of Medicine, Kaohsiung Medical University, Kaohsiung, 80708 Taiwan; 5 Waseda Research Institute for Science and Engineering, Waseda University, Tokyo, 169-8555 Japan

**Keywords:** Imaging, Sensors and probes, Chemical modification, Enzyme mechanisms, Proteases

## Abstract

Understanding cellular signaling flow is required to comprehend living organisms. Various live cell imaging tools have been developed but challenges remain due to complex cross-talk between pathways and response heterogeneities among cells. We have focused on multiplex live cell imaging for statistical analysis to address the difficulties and developed simple multiple fluorescence imaging system to quantify cell signaling at single-cell resolution using Förster Resonance Energy Transfer (FRET)-based chimeric molecular sensors comprised of fluorescent proteins and dyes. The dye-fluorescent protein conjugate is robust for a wide selection of combinations, facilitating rearrangement for coordinating emission profile of molecular sensors to adjust for visualization conditions, target phenomena, and simultaneous use. As the molecular sensor could exhibit highly sensitive in detection for protease activity, we customized molecular sensor of caspase-9 and combine the established sensor for caspase-3 to validate the system by observation of caspase-9 and -3 dynamics simultaneously, key signaling flow of apoptosis. We found cumulative caspase-9 activity rather than reaction rate inversely regulated caspase-3 execution times for apoptotic cell death. Imaging-derived statistics were thus applied to discern the dominating aspects of apoptotic signaling unavailable by common live cell imaging and proteomics protein analysis. Adopted to various visualization targets, the technique can discriminate between rivalling explanations and should help unravel other protease involved signaling pathways.

## Introduction

Tools are continually being developed to elucidate the mechanisms involved in cell fate through promotion or suppression of intracellular signal transduction under heterogeneous conditions^1^. Difficulties are encountered due to the complexity of cellular signaling networks and variations in the response of cells. To investigate spatiotemporal cross-correlations and causality between the signals in the single cell, effective tools to quantify multiple signaling activities are needed more. In order to gain generalizable insights into signaling flow beyond cell heterogeneity, population-level evaluation of diverse signals through detailed single cell observation is required. Thus, to extract universal features through the cell population, high-content imaging systems have been devised^2,3^. The specific devices with molecular sensors for multiple common cellular phenomena and statistical approaches have enabled researchers to simultaneously track diverse cellular events^4–6^. The devices can be operated to get quantified image of the cellular events using routine analysis. At the point, tracking data are not always obtained according to the signaling flow because commercially available efficient molecular sensors for routinized imaging are focused on usual metabolic activities, ROS fluctuation, cell volumes, and viabilities, and few device-optimized sensors for the specific cellular event are readily available. Other fluorescence spectroscopy using microscopes, flow cytometers, and microfluidic devices has different potential to advance live-cell imaging^7–9^. However, it is still difficult to balance specific, sensitive, immediate, and quantitative spatiotemporal detection of focused steps of signaling pathways simultaneously and their expedite population level analysis^2,3,10–12^.

Notably, the elaborated molecular sensors for the imaging platform warrant further improvement. Many genetically encoded Förster Resonance Energy Transfer (FRET)-based molecular sensors have been accepted for the quantitative detection of cellular signaling, but most of these were not for entirely applicable for parallel use because CFP-YFP FRET pairs were primarily employed due to high sensitivities for target phenomena not requiring complicated calibration on the points of cross-excitation through for quantitative FRET measurements^13–18^. Although some groups have developed red-shifted FP-based molecular sensors^19,20^ or supplant combinations with even not use of CFP-YFP’ combinations^21,22^ for multiplexed FRET pairs imaging, there seemed to be some difficulties in spectral overlapping of fluorescent windows between FPs, relative low quantum yields of orange, red and infrared FPs, and dual excitations necessity for individual FRET pairs. To complement those complexities and increase the accuracy of imaging, combined use of other fluorescent measuring approaches have been reported^23–25^. Besides, it is not easy to change imaging targets, once engineered constructs have been specifically optimized. Those situations might keep multiplexed FP-based FRET sensing away from versatile routine imaging works. On the other hand, synthetic molecular sensors with differentiating fluorescence properties may have proven useful in the concurrent detection of target molecules^26–28^; however, this approach would rather be good at monitoring amount of substances or changes and takes some effort to detect enzymatic reactions for specific cellular signaling^29^. Therefore, to expand the fluorescence spectroscopic approach by picking the best of both types of sensor molecules, we developed non-invasive FRET-based chimeric molecular sensors comprised of a fluorescent protein as the donor molecule and a fluorescent organic dye as the acceptor molecule (bioprobes). In the case of protease activity sensing, initial FRET was immediately cancelled through proteolysis^30^ (Fig. 2A). Since the FRET-based chimeric molecular sensors were not bound by common Förster distance rules^31–33^, the combined fluorescent donor and acceptor molecules emitted visible or near-infrared light with a large apparent Stokes shift at the point of spectroscopic resolution irrespective of the target^34,35^. This enables researchers to select appropriate FRET pair combinations adjusted for their equipment mounting unique settings. Additionally, the high intramolecular FRET efficiencies provided some advantages upon fluorescent signal acquisition originating from most bioprobes. We were able to calculate net FRET efficiency without any laborious compensation, which could be easily approximated from the ratio of donor to acceptor fluorescence intensity at a single excitation setting^34,35^. Consequently, bioprobes for various targets can render normalized FRET signals because of simple measurements of net FRET efficiencies. Preparation processes of bioprobes required no tricky handling even for substitution of imaging target^36^. As introducing obtained bioprobes into cells either with a protein delivery system or direct mixing into culture supernatant was easy and efficient to adapt quantity ratio of each bioprobes introduced considering their sensitivities, this strength, but not weakness,　facilitated to repeat the observation of multiple bioprobes simultaneously and acquire corresponding imaging promptly. Imaging system using our bioprobes, therefore, might be consistent with quantitative multiplexed live imaging and population level analysis.

Programmed cell death, is a diverse, critical phenomenon in multicellular organisms^37–39^. As apoptosis is a representative type of programmed cell death, programmed apoptotic cell death, programmed non-apoptotic cell death, and non-programmed cell death (necrotic cell death) have been convincingly classified by^40^. By avoiding apoptotic cell death, many cancer cells accomplish abnormal growth that can be life-threatening^41–45^. Although the ambiguous regulation mechanisms arising from complex cell death signaling networks still give rise to controversies over hallmarks and details of classification, cysteine-aspartic proteases, caspases, a family consisting of more than 10 member enzymes, play essential and distinct roles mutually related for diverse types of cell death to determine cell fate as programmed or non-programmed cell death or cancellation of cell death^46–48^. As the caspase family enzymes have similar activation mechanisms and digestible substrate amino acid sequences but complex interrelationships due to different functions in diverse types of cell death, caspases are an ideal target to demonstrate the utility of our imaging-derived statistics using bioprobes to clarify selection mechanisms for cell death.

For this study, we thus applied the customizable fluorescence imaging system to detect simultaneous distinctive caspase activations and estimated the regulatory relationship through analysis of enumerated individual cell data. By applying this to the evaluation of recognized steps involving caspase-9 and -3 for the decision of apoptosis execution or not^48–51^, we could give a minute explanation of mutual correlation modes for caspases extracted regarding time and activity unlike in the case of simple orders for signaling flow.

## Results

### An instance of the bioprobe-based fluorescence imaging system

**Figure 1:**
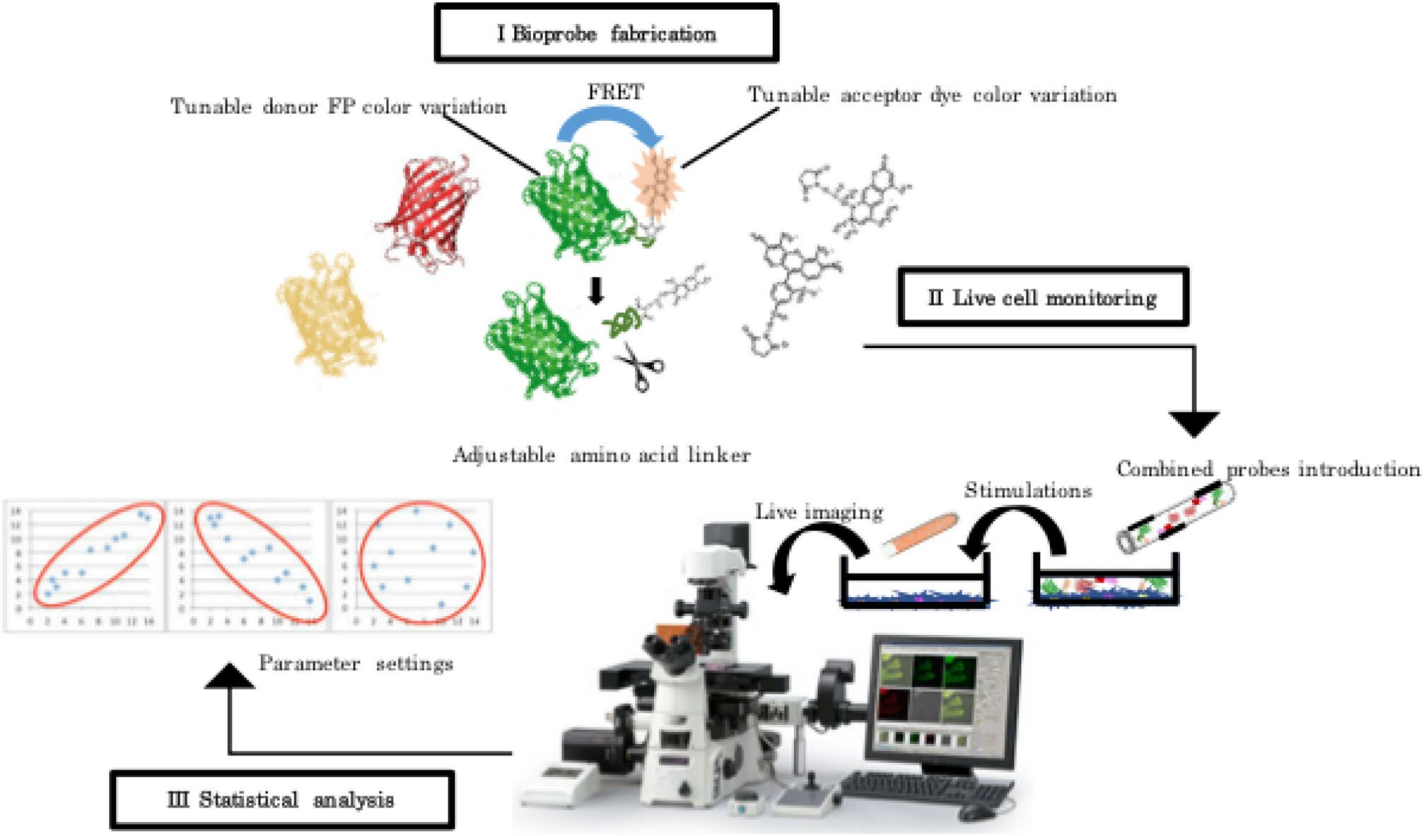
Custom-made fluorescence imaging system consisting of 3 parts: (I) Bioprobe fabrication characterised by tunable components. (II) Live imaging characterised by equipment adjustability and simple approaches. (III) Statistical analysis characterised by flexible parameter settings.

First, we determined FRET-based chimeric molecular sensors (bioprobes) to implement the imaging system, as shown in Fig. 1 (detailed bioprobe manufacturing processes are depicted in Fig. S1). Our imaging system features good adjustability of bioprobes consisting of donor fluorescent proteins, target recognition parts and acceptor chemical dyes. We have summarized the composition of some bioprobes for proteolytic reactions extracted from previous works in Table 1 to exhibit the adjustability. We have already confirmed our bioprobes functioned equivalently in HeLa and Jurkat^34^. We demonstrated our bioprobe implementation versatility using additional cell lines (human epidermal keratinocytes and mouse embryonic fibroblasts) upon apoptosis inductions here (Fig. S2). Cell population introduced bioprobes shifted altered populations with emission properties of FRET decreases in response to apoptosis induction. The manufacturing processes were fixed and demonstrate an uncomplicated and robust arrangement^34,52^. Therefore, fluorophores can be coordinated according to the specifications of the equipment for fluorescence resolvability and sensitivity. We have shown that practical FRET efficiencies for most adapted bioprobes can be calculated from the raw donor to acceptor fluorescence intensity values without the need for compensation. Two or three separate bioprobes, optimised for the observation system, can thus be simultaneously employed for individual event monitoring. Bioprobes can also be controllably and efficiently delivered into cells. Hence, corresponding observations of imaging data were repeatedly collected for statistical processing. Upon processing data, adequate indices were utilized to assess normalized enzymatic activities in this study. A novel imaging system that enabled simultaneous visualization of proteolytic activities using bioprobe technology and a statistical approach was thus established.

**Table 1:** Bioprobes already reported. Composition of some bioprobes previously generated were extracted to show high adjustabilities.

Donor FP	Acceptor dye	Target	Ex./Em (nm)	Reference
GFP	Alexa Fluoro-532	caspase-3	532/553	^21^
GFP	Alexa Fluoro-546	caspase-3	556/575	^25^
GFP	Alexa Fluoro-555	caspase-3	555/565	^25^
GFP	Alexa Fluoro-555	caspase-9	555/565	^26^
GFP	DyLight 680	caspase-9	692/712	^26^
GFP	Alexa Fluoro-594	caspase-3	590/617	^25^
GFP	Alexa Fluoro-750	caspase-3	749/775	^26^
GFP	Eosin	caspase-3	524/544	^21^
GFP	Eosin	trypsin	524/544	(a)
GFP	Tetrametyhlrhodamine	trypsin	540/567	(b)
RFP	Alexa Fluor-633	caspase-3	632/647	^25^
RFP	Alexa Fluor-647	caspase-3	650/665	^25^
RFP	Alexa Fluor-660	caspase-3	663/690	^25^
RFP	Alexa Fluor-680	caspase-3	679/702	^25^

(a) Suzuki, M., Ito, Y., Savage, H.E., Husimi, Y. & Douglas, K.T. Biochim Biophys Acta. 1679, 222–229 (2004).

(b) Suzuki, M., Ito, Y., Savage, H.E., Husimi, Y. & Douglas, K.T. Saitama Univ. Bulletin Dept. of Eng. 37, 29–37 (2004).

### Adjustment of the bioprobe for caspase-9 in live-cell imaging

**Figure 2:**
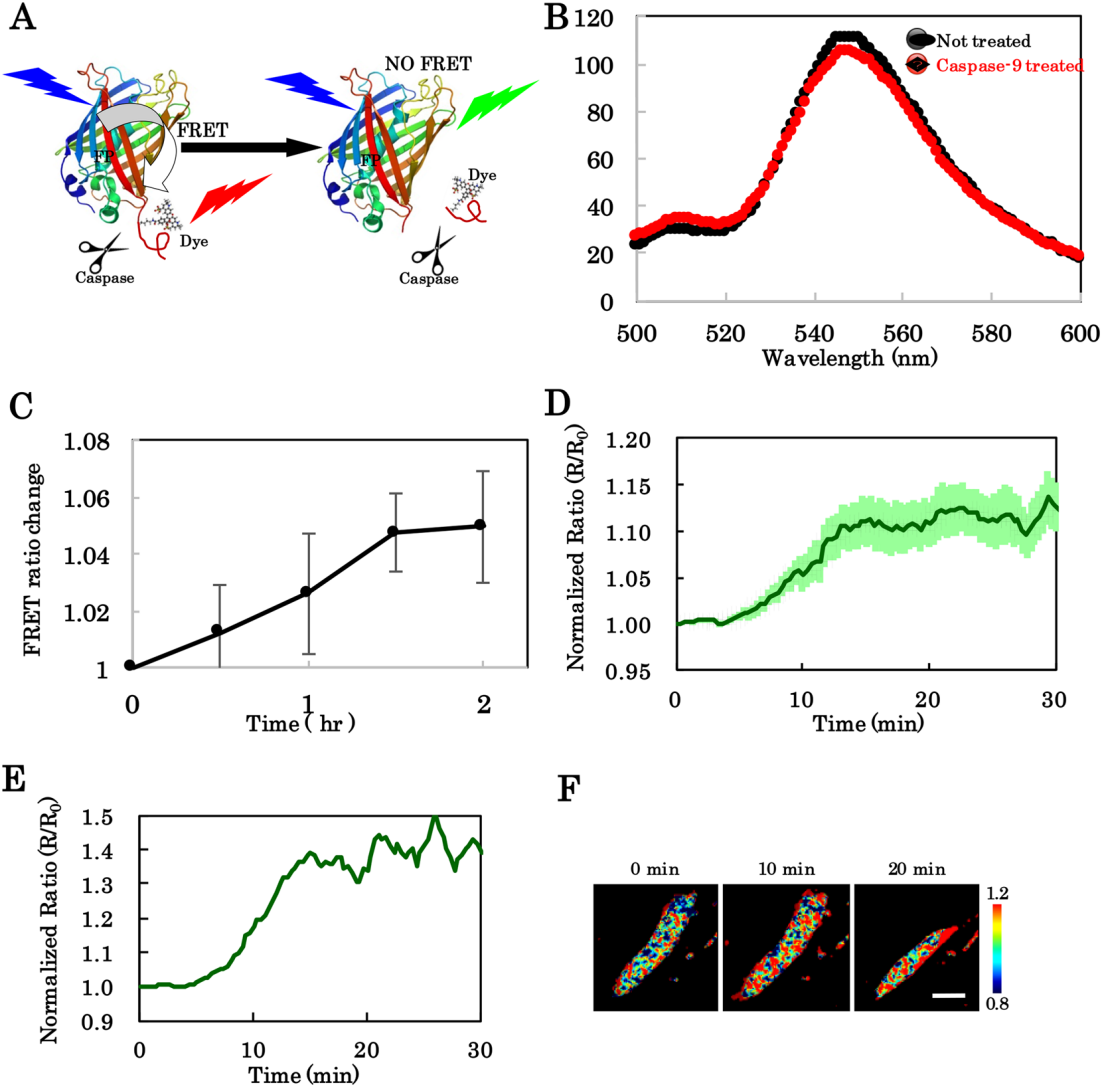
Functional mechanism and detection properties of caspase-9 with FRET-based chimeric bioprobe. (**A**) Schematic of FRET-based sensing upon caspase treatments (**B**) Emission profile with or without caspase-9 treatments of bioprobe consisting of donor molecule (GFP) and acceptor molecule (Alexa Fluor 532). (**C**) Time-dependent FRET ratio change of the bioprobe after capase-9 treatment in vitro. (n = 3, values represent mean ± SD) (**D**) Averaged time-dependent FRET ratio change of the bioprobe after introduction into HeLa cells after apoptosis induced with TNF-α and cycloheximide at 0 min. (n = 17 cells). Error bars indicate SEM. (**E**) Representative time-dependency of the bioprobe response extracted from (**D**). (**F**) Pseudo-colour images for FRET ratios observed at three different time points from €.

We identified an optimal bioprobe for caspase-9 considering the measuring device specifications. In a previous study, measurements of bioprobe fluorescence lifetime were employed to estimate caspase-9 activity inside the cell^35^, due to the more indistinct activation of caspase-9 compared with that of caspase-3^53^. In the current study, we aimed to achieve an improved bioprobe sensitive to ratio changes during fluorescence microscopy. First, to improve the foldability of the mutant GFP, we restored spacer amino acid sequences inserted around the caspase-9 recognition site (LEHD) to the original C-terminal sequence of green fluorescent protein (GFP), which was deleted in the previous version of the bioprobe for caspase-9 due to the insertion. Changes compared to the previous version in detail was shown in Fig. S1. Next, Alexa Fluor 532 was chosen as the acceptor dye based on sufficient wavelength separation for the fluorescence filter and laser settings, as shown in Fig. S3B. Emission spectra of the bioprobe with or without caspase-9 treatment in vitro are shown in Fig. 2B. The time-dependent changes in FRET ratio upon caspase 9 treatment were examined (Fig. 2C). The GFP-Alexa Fluor 532 bioprobe had high FRET efficiency but weak reactions; however, based on previous observations, the bioprobe was anticipated to be sufficiently sensitive to monitor caspase performance inside cells, which have a suitable environment for enzymatic reactions^30^. The bioprobe was introduced into HeLa cells to assess its responsiveness to caspase-9 activity in apoptosis-induced cells. Strong signals were observed after bioprobe introduction, accompanied by cell volume shrinkage following apoptotic initiation using TNF-α through programmed cell death receptors as extrinsic^39^ and cycloheximide as intrinsic stimuli^54^. FRET ratio changes of the bioprobe were normalised over time. An averaged time-course and representative images corresponding to conventional microscopic experiments for spatial information on cells are shown in Fig. 2D–F. The results indicated that an optimal bioprobe for detecting caspase-9 activity in fluorescence microscopy had been found.

### Caspase-9 and -3 activation imaging in apoptotic cells

To detect caspase-3 function in cells, a red fluorescent protein (RFP)-based bioprobe conjugated with Alexa Fluor 660 was chosen. This bioprobe was previously validated by examining the behaviour of cell populations during induction of apoptosis using flow cytometry. We verified that this bioprobe had sufficient ability to detect caspase-3 activity by set fluorescence microscopy (Fig. S3C). Both GFP-Alexa Fluor 532 and RFP-Alexa Fluor 660 bioprobes could be excited at 440 nm due to broad excitation spectrum of the RFP used (Fig. S3B). Moreover, relatively sharp excitation spectrum of Alexa dyes minimized direct acceptor excitation, indicating that this pairing with matching fluorescence properties would system well under experimental conditions (Fig. S3B). In addition, linear spectral unmixing was found to effectively enhance signal separation (Fig. S3D).

**Figure 3:**
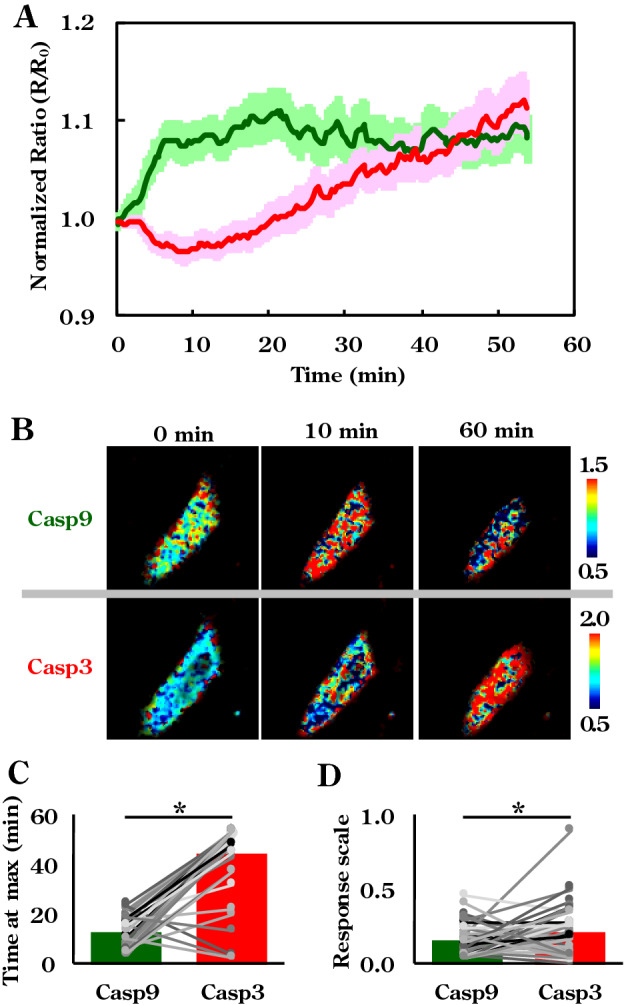
Simultaneous assessment of caspase-9 and caspase-3 activity in single cells undergoing apoptosis. (**A**) Averaged raw activity of individual caspases (green: caspase-9, red: caspase-3) upon apoptotic stimulation. (n = 44 cells, error bars indicate SEM) (**B**) Pseudo-colour images of the response patterns of caspase-3- and caspase-9-sensitive bioprobes at three corresponding time points. (**C**) Averaged and individual values of times at maximum activity for respective caspases. (**D**) Averaged and individual values of response scale for respective caspases. (n = 44 cells, bar graphs indicate the average, dots and bars indicate the values of each cell, * indicates *P *< 0.05 by t-test).

Activation of caspase-9 and -3 in each cell was then simultaneously observed using the paired bioprobes. After introduction of concentration-modulated bioprobes set into HeLa cells, apoptotic pathways were provoked with both TNF-α and cycloheximide, as before^30^. We observed cell shrinkage in > 30 cells via fluorescence microscopy to estimate average time-dependent changes in normalised FRET ratios just after induction of apoptosis. As the bioprobes behave as pseudo substrates for target enzymes, thus competing with native substrates, we compared the behaviours of the paired bioprobes (Fig. 3A) with that of each individual bioprobe in separate experiments using fluorescence microscopy (Fig. 2D and Fig. S3C) and with results of prior studies^48–51^. The paired, just as the individual single molecular sensors, were shown to respond to apoptotic stimulation without distorting the signaling flow (representative images processed with pseudo-color indication are displayed in Fig. 3B). Activation of caspase-9 seemed to occur around the periphery of the cell membrane and dispersed over the cytosolic area quickly, whereas caspase-3 activation was initiated evenly within the cytoplasm and spread to the nucleus, followed by shrinkage of the cell. These spatial activation profiles were consistent with previous studies on apoptotic phenomena^31^, in which activated cytosolic caspases such as caspase-8 and -9 mediated activation of downstream caspase-3, followed by nuclear translocation of caspase-3 to perform the digestion of crucial substrates, which is key to apoptosis^48–50^. Interestingly, some individual response patterns differed substantially from the average pattern due to cellular heterogeneity (five distinctive patterns, pattern I to V, are illustrated in Fig. S4A–E). The average duration to reach the maximum ratio during observation (time at max) and response scale derived from caspase-3 activation were longer and larger than those derived from caspase-9 activation. These data are consistent with conventional protein expression analysis. However, there seemed to be no clear correlation between individual time at max and response scale derived from caspase-3 and -9 in individual cells (Fig. 3C,D). Conversely, single cell data delivered distinct response patterns for analyzing the population in detail.

### Elucidation of underlying regulation between caspases

**Figure 4:**
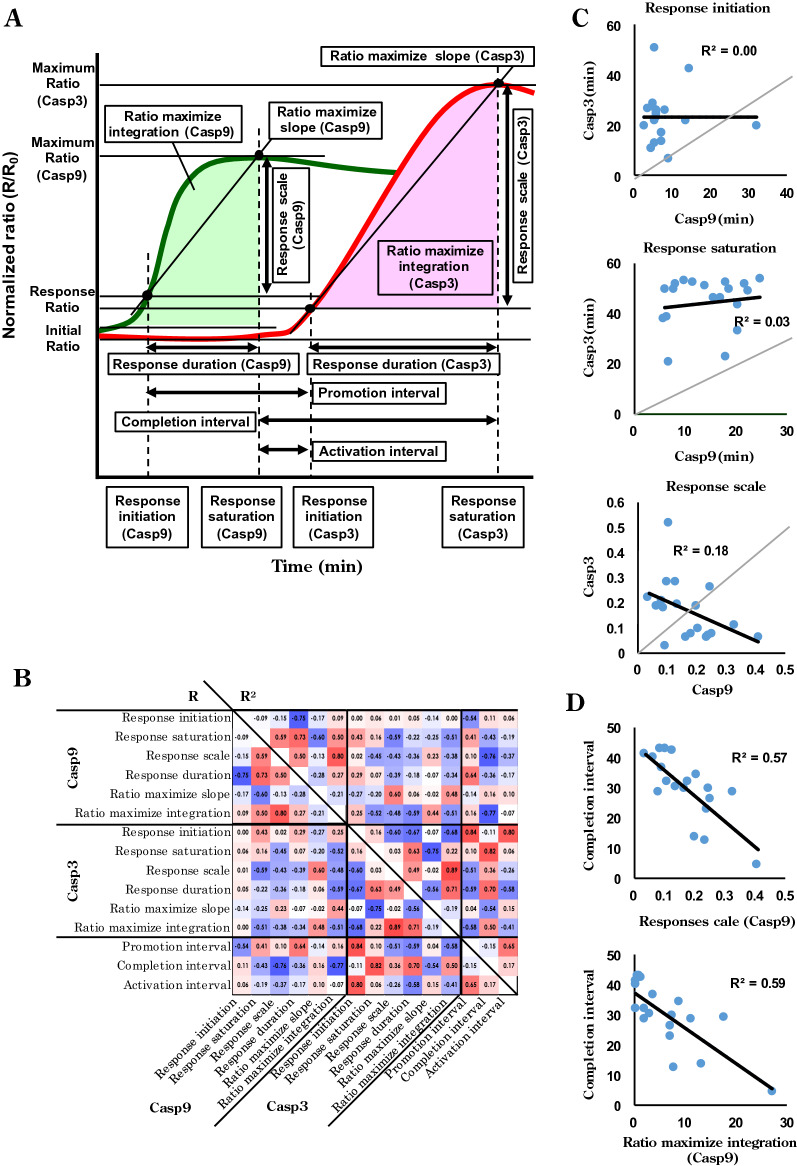
Indices defined to characterise caspase behaviour and statistical analysis of caspase-9 and -3 activation correlations. (**A**) Schematic of each caspase (green: caspase-9, red: caspase-3) activation profile categorising response strengths (response scale, maximum ratio, ratiomaximise integration), times (response initiation, response saturation, response duration, promotion interval, activation interval, completion interval), and rates (ratiomaximise slope). (**B**) Heatmap of caspase-9 and -3 activation correlations showing correlation coefficients (R: lower half) and coefficients of determination (R^2^: upper half) of all indicator combinations with positive correlations in red and negative ones in blue. (n = 19 cells) (**C**) Correlations between ratio initiation, ratio saturation, and ratio scale of caspase-9 and -3. (**D**) Correlations between completion intervals and response scale or ratiomaximise integration of caspase-9.

To perform detailed statistical analysis on the data collected from the paired bioprobes shown in Fig. 3A, authentic and characteristic indices and terms were introduced as follows: response initiation, FRET ratio change (activity elicitation) detection time; response saturation, finished FRET ratio change (activation attainment) after apoptotic stimulation; response scale, difference between initial FRET ratio and maximum; promotion, activation, and completion intervals or response duration; intervals of response initiation and/or saturation, respectively; ratio maximize slope and ratio maximize integration, acceleration rate and total of enzymatic reactions, respectively (Fig. 4A). All data applicable to our indices were extracted, and correlations were represented as a heatmap (Fig. 4B). The heatmap patterns among the respective caspases exhibited homologous features, a result that was predictable based on the self-regulation of this family of enzymes. The stronger intra-correlation of caspase-3 compared to caspase-9 in corresponding areas may have resulted from differences in parts of the signaling flow and previously identified activation processes^48–50^. As noted for Fig. 3C,D, simple links may not exist between response initiation, response saturation, and response scale of the two caspases (Fig. 4C) or between the indices as indicated by the scatter plots (Fig. S4F). Strong correlations were observed between completion interval and response scale or ratio maximize integration for caspase-9, demonstrating that the total outcome for caspase-9 governs the performance of caspase-3 (Fig. 4D). Assuming this regulation, it seems reasonable that the completion interval is less correlated with the ratio maximize slope of caspase-3 (Fig. 4B). More precisely, it was shown that the upstream protease, caspase-9, controlled times to saturation of the downstream protease, caspase-3, activity depending on total caspase-9 activity, but not the reaction rate of caspase-9. This indicates that activated caspase-9 can continuously activate caspase-3 until sufficient activity of caspase-3 will be attained.

## Discussion

We identified connections, in the final stages, of the apoptotic signaling network in HeLa cells using novel imaging system based on bioprobe technology. We demonstrated that the activity of the inclusive initiator protease, caspase-9, in our setup has a negative correlation with the time required for saturation of the executioner protease, caspase-3, activity. This suggests that pro-caspase-3 activation through cleavage by active caspase-9 and apoptosis execution carried out by the activated caspase-3 digestion of its target proteins may be supposed to complete within a time frame that makes further impactful control processes unnecessary. The elucidation can consistent with previous study for caspase cascades and the functions of caspase-9 and -3: it would be necessary for apoptotic progress to accumulate sufficient amount of activated caspase-3 inside the nucleus promptly to digest a target, Poly(ADP-ribose) polymerase PARP-1, a key protein for the DNA damage response to choose repair pathways^55–57^ and after that manage cell reponses as apoptotic or necrotic cell deaths, or survival^58,59^.

From our comprehensive findings, it appears that accumulation of mature caspase-3 through linear caspase-9 activation and its prompt nuclear translocation will determine the time it takes to cleave and inactivate PARP-1. Appropriate time-dependent digestion of the target will give cells a choice of cell response to accomplish apoptotic cell death^60,61^. It can be supportive observation shown in Fig. 3B for caspase-3 activation to initiate evenly within the cytoplasm and spread to the nucleus.

Since all statistical investigations in this paper were carried out only for apoptotic dead cells following atypical responses elimination (Fig. 3A), this procedure may collect cell populations sensitive to PARP-1 dependent DNA repair, such as cells in the S phase of the cell cycle or the checkpoints during S phase, leading to immediate activation of caspase-3 after apoptosis induction^62–64^. These interpretations were indicated by our imaging based statistical analysis for quantitative temporal detection of two steps in the apoptotic signaling pathways in single cells. Tracking caspase-3 activities shown in Fig. 3A and Fig. S3C, our bioprobes behaviors after start sensing appeared not to be corresponding to theoretical activities, i.e. though bioprobes were supposed to show only increase in FRET ratios upon proteolytic sensing, bioprobes responded to apoptosis induction oppositely　during several tens of minutes. The decrease in FRET ratios might be caused by emission alterations of FPs resulting from drastic environmental exchanges inside cells such as pH, ionic strength, viscosity etc. As similar phenomena have already been discussed using FP-based live imaging^23^, we recognized it applied to this case as well but with no major impediment. Our technique could thus reveal crucial detailed information on time dependent cellular events despite of the complex signaling flow^65^. We note that the simple analysis shown in Fig. 3C,D, suggests that averaging data from a reasonable number of cells obtained by measurements using bioprobes provide results comparable to those of established experiments exploring averaged expression or modification profiles of key molecules in cell lysates^66^. Unlike what can be obtained from averaging data, even simple enumeration of unprocessed data indicated the existence of distinct sub-populations (Fig. S4A–E). These findings may have arisen from conventional fluctuations, dispersed apoptotic potential due to an epigenetic background, and cellular conditions at various cell cycle stages^41^. Moreover, other functions of caspases might be invoked through interactions with as yet unspecified substrates or regulator molecules relevant to apoptosis or other processes. Atypical but frequent responses could lead to the discovery of new types of programmed cell death.

Imaging using different indices with readapted bioprobes for equivalent experiments recreated to classify the response patterns more closely or imaging using the same indices with bioprobes improved in terms of sensitivity through sequence arrangements around the enzyme recognition sites may reveal the apoptotic pathway in further detail.

Our customizable fluorescence imaging system can also be modified to demonstrate various signaling pathways other than the apoptotic one. Confirmed techniques via construction of chimeric bioprobes are genetically and chemically expandable. Similar developed FRET-based molecular sensors for other families of proteases, other types of hydrolase, pH alterations^67^, and intracellular congestion can be arranged into appropriate bioprobes (unpublished data) and integrated into this imaging system. Based on sufficient separation of wavelengths in visible light, it might be feasible to monitor three different protease activations with distinct bioprobes, providing better insight into the complex regulatory mechanisms for signaling flow. In addition, bioprobes could be aimed at organelles by fusing a localization peptide to the fluorescent protein, allowing visualization of space specific signaling. It is anticipated that comprehensive and statistical analysis of individual cell- and population-level relationships for well-chosen observable phenomena will further resolve their functional potential and the driving forces of cellular events. This should result in the identification of previously unknown branched pathways or reconsideration of known signaling pathways.

Significantly, this technology is neither restricted by combinations of bioprobes nor the cell type into which they are introduced. However, there might be some difficulties for cell types with less efficient bioprobs uptake or in vivo imaging. Bioprobe construct expansions combined with genetically encoded FPs and their labeling by click chemistry inside cells or in vivo could develop this technology applicable^68^. Therefore, the imaging system proposed in this study could also be useful for effective screening or discovery of therapeutic drugs.

## Materials and methods

### Plasmid construction for caspase-9 and -3 activity detection

Inverse polymerase chain reaction (iPCR) was used to construct a plasmid containing the reporter genes for GFP and to detect caspase-9 activity. This plasmid was based on the pUV5cas12-1 plasmid and its caspase-9 sensing capabilities were verified through flow cytometry^35^, where we altered amino acid sequences around the caspase-9 recognition sequence, LEHD, from SGSSGIT LEHD GTCELYK to ITHGMDELYK LEHD TC just after the C-terminus of the GFP core (Fig. S3A). PCR performance was confirmed by sequencing, and the resulting plasmid was named pUV5casM-22. For caspase-3 detection, we prepared an RFP-based construct as previously described, which we named pT3castag^34^.

### Fluorescent protein isolation

GFP and RFP variants were isolated and verified as previously described^34^. The two plasmids described above (pUV5casM-22 and pT3castag) were transformed into *Escherichia coli* BL-21(DE3) (Merck Biosciences, Darmstadt, Germany). Each bacterium obtained from a single colony was grown at 37 °C in lysogeny broth (LB) medium containing 75 μg/mL ampicillin for 12 h. Subsequently, an aliquot of the culture medium was diluted 10 × with fresh LB medium supplemented with 75 μg/mL ampicillin, and cultured at 28 °C for 48 h. The bacteria were harvested and lysed with bacterial protein extraction reagent B-PER II (Thermo Fisher Scientific Inc., Waltham, MA, USA), according to the manufacturer’s instructions. The lysates were centrifuged at 15,000 rpm for 15 min at room temperature. The supernatant was applied to Ni^2+^-NTA resin (Qiagen, Düsseldorf, Germany), and the His-tagged fluorescent proteins were isolated using affinity chromatography according to standard procedures for purification of histidine tag-fused proteins. The recovered protein was reconstituted in phosphate-buffered saline (PBS) and subjected to sodium dodecyl sulfate–polyacrylamide gel electrophoresis (SDS–PAGE) to determine purity and oligomerisation. The purified protein was further quantified and qualified by spectrophotometry, with absorbance at 280 nm and 488 nm to determine the GFP variant, and 280 nm and 565 nm to determine the RFP variant.

### Preparation of bioprobes

Bioprobe preparation was conducted as previously described^30^. Briefly, the purified proteins were chemically modified with fluorescent dyes, Alexa Fluor 532 for the GFP derivative, and Alexa Fluor 660 for the RFP derivative (both from Life Technologies Corp., Carlsbad, CA, USA). The proteins were dissolved in PBS (10 μL) containing 1 mM dithiothreitol (DTT) to give a final concentration of 10 μM, which was then incubated for 10 min at room temperature. DTT was removed by gel filtration (NICK column, GE Healthcare UK Ltd., Chalfont St Giles, UK). An aliquot of the elutant (400 μL) was immediately mixed with 2 μL fluorescent dye in dimethyl sulfoxide (DMSO) solution (2 mg/mL), and the mixture was incubated at 37 °C for 4 h. Excess dye was removed through gel filtration on a NICK column equilibrated with PBS. The fluorescence spectrum was measured at an excitation wavelength of 488 nm (for GFP derivatives) or 565 nm (for RFP derivatives) using a Shimadzu RF-5300PC Spectro fluorophotometer (Shimadzu, Kyoto, Japan). Because the FRET efficiency is defined as the probability of donor molecules transferring fluorescent excitation energy to acceptor molecules, and not as the ratio of donor to acceptor fluorescent excitation energies, we compared the corresponding values to assure relative equivalence. Therefore, the term FRET ratio in this study refers to approximate FRET efficiencies to normalise caspase activities.

### Determining Caspase-9 activity assay with bioprobes in vitro

We determined caspase-9 sensing in vitro according to a previously established approach^26^ with the Alexa Fluor 532- UV5casM22 conjugate bioprobe. Here, 4.0 units of caspase-9 (Abcam, Cambridge, UK) were added to 40 μL of 2.0 μM bioprobe solution reconstituted in an assay buffer containing 50 mM 2-[4-(2-hydroxyethyl)piperazin-1- yl]-ethane sulfonic acid (HEPES), 50 mM NaCl, 0.1% 3-[(3- cholamidopropyl)dimethylammonio]-1-propanesulfonate(CHAPS) detergent, 10 mM DTT, 5% glycerol, and 10 mM ethylenediaminetetraacetic acid (EDTA). The solution was then incubated at 37 °C for 2 h. Aliquots of the solution were removed and diluted two-fold with assay buffer at each time point, and caspase-9 specificity was measured by assessing changes in FRET ratios with the JASCO FP-8500 Spectro fluorophotometer (JASCO Corp., Tokyo, Japan).

### Introduction of bioprobes into cells

Introduction of the bioprobes into cultured cells was performed as previously described^30^. HeLa cells were cultured in a 35-mm dish with HyClone Dulbecco's modified Eagle's medium (DMEM)/high glucose culture medium (Thermo Fisher Scientific) with 10% foetal bovine serum (FBS) at 37 °C under an atmosphere containing 5% CO2. The cells were washed 3 × with 0.5 mL Hank’s Balanced Salt solution (HBSS), of which 0.513 mL was added to the culture. An aliquot of the bioprobe mixture (24 μM for the RFP-Alexa Fluor 660 conjugate or 12 μM for the GFP-Alexa Fluor 532 conjugate) in PBS (37 μL) was added to a tube covered with a dry film of BioPORTER reagent (Gene Therapy Systems, San Diego, CA, USA). The resulting mixture was hydrated for approximately 10 min at room temperature. This solution was then added to the prepared HeLa cells, as described above. The cells were incubated for 4 h at 37 °C under an atmosphere containing 5% CO2. The residual bioprobes and BioPORTER-protein conjugates were removed by washing the cells with culture medium without FBS. The cells were then incubated for 1 h in culture medium supplemented with 10% FBS. Subsequently, the culture medium was replaced with serum-free culture medium containing apoptosis-inducing reagents.

In case of direct incorporation of bioprobes into cells, HeLa, NIH3T3 and FEPE1L-8 cells were washed 3 × with fresh culture medium supplemented with 10% foetal FBS and added with aliquots of which containing 5 μM corresponding bioprobe for 12 h incubation at 37 °C under an atmosphere containing 5% CO2. The residual bioprobes were removed by washing the cells with culture medium without FBS. Immediately after this, apoptosis induction could be done by replacement with serum-free culture medium containing apoptosis-inducing reagents. Just after uptake of Alexa Fluor 532- or Alexa Fluor 555- UV5casM22 conjugate bioprobes into HeLa cells in a 35 mm dish, recovered cells were analysed using flow cytometry to check introduction efficiencies of bioprobes as shown in Figure S4. The culture supernatant with the bioprobe was removed and rinsed with 250 μl of 0.2 mM EDTA. The chelating solution was removed and incubated with 250 μl each of Accutase and Accumax cell detachment mixtures (Innovative Cell technologies, San Diego, CA, USA) for 10 min at 37. The detached mixtures with the detached cells were stored as recovered cell solutions. Trypsin (0.25%, 250 μl) was then added to the culture dish for complete cell detachment. The resulting solutions with cell debris were combined with those previously stored and the whole solutions were subjected to flow cytometry analysis using a SONY SH800Z (SONY Corp., Tokyo, Japan). The data obtained were processed by FLOWJO software (Becton, Dickinson and Company, San Jose, CA, USA). HeLa cells and NIH3T3 cells were kindly provided from department of life science, graduate school of science and engineering, Saitama university and maintained to avoid mycoplasma contaminations. FEPE1L-8 which have been transfected　HFKs with transforming genes from human papilloma virus were kindly donated by Dr. W. G. Carter (Fred Hutchinson Cancer Research Center, Seattle, WA)^69^.

Although highly efficient and stable bioprobe incorporations into HeLa cells by direct introduction were observed and resulted in cell populations with consistent fluorescence intensities originating from bioprobes maintaining FRET conditions (Fig. S5) and proper responses of bioprobes (FRET cancellation) consisting other combinations for donors and acceptors were obtained using other cell types (Fig.S2), we applied BioPORTER reagents in this study for strict control of quantity ratios of bioprobes introduced into cells.

### Apoptosis induction

Induction of apoptosis in HeLa cells was conducted as previously described^30^. HeLa cells with internalised bioprobes were incubated in HyClone DMEM/High Glucose culture medium supplemented with 10% FBS for 1 h to confirm bioprobe distribution inside the cells. The medium was then replaced with FBS-free culture medium. 100 ng/mL and 1.0 mg/mL of tumour necrosis factor alpha (TNF-α) and 4-{(2R)-2-[(1S,3S,5S)-3,5-dimethyl-2-oxocyclohexyl]-2-hydroxyethyl}piperidine-2,6-dione (cycloheximide) were added to the fresh medium, respectively. The treated cells were subjected to microscopic observation.

### Live cell imaging with fluorescent microscopy

Fluorescence imaging was performed at room temperature using an FV-1000 confocal microscope (Olympus, Tokyo, Japan) with a × 40 oil immersion objective lens. To simultaneously monitor caspase-9 and -3 activity, both bioprobes were introduced into HeLa cells. Both GFP and RFP were excited at 440 nm from a diode laser through a dichroic mirror (405–440/515), and fluorescence was separated by three dichroic mirrors (510 nm, 560 nm, and 640 nm) and detected by four photomultipliers through suitable band path filters; 460–510 nm for GFP, 520–550 nm for Alexa 532, 575–620 nm for RFP, and 655–755 nm for Alexa 660, as shown in Figure S2B. Fluorescence images were obtained every 20 s.

### Image analysis

Although the fluorescence signal was separated by dichroic mirrors and band-pass filters as described above, each obtained fluorescence signal contained fluorescence leakage from other chromophores (Fig. S3B). To separate these fluorescence signals, linear unmixing was applied in Matlab (MathWorks, Natick, MA, USA) as previously described^21,70^. The separated signals were analysed using Aquacosmos software (Hamamatsu, Shizuoka, Japan). Each region of interest (ROI) was located in an area containing the whole cell body, and the average fluorescence intensity was obtained. The fluorescence ratio of GFP to Alexa 532 corresponded to caspase-9 activity and that of RFP to Alexa 660 corresponded to caspase-3 activity.

### Statistical evaluation

Fluorescence ratios were normalised to initial values, and the characteristic values of the response of each cell were detected as shown in Fig. 4A. Response initiation was detected as supra-threshold periods (average + 3 × SD of 10 frames before FRET ratio increase). Correlations between parameters were calculated and displayed in a heatmap using Microsoft Excel 2016.

## Supplementary Information

Supplementary Information.

## Data Availability

The datasets generated or analysed during the current study are available from the corresponding author on reasonable request and in the DDBJ repository, https://www.ddbj.nig.ac.jp/services/index-e.html. Accession numbers and ID as follows: LC710244: 627c85173a01a50064a74723.GFP-cas3-2; LC710245; 627c85173a01a50064a74723.GFP-cas9-2; LC710246: 627c85173a01a50064a74723.GFP-Ncas9-1; LC710247: 627c85173a01a50064a74723.GFP-Ncas9-2; LC710248: 627c85173a01a50064a74723.RFP-cas3-2.
