# Supplementary material for: Live imaging of apoptotic signaling flow using tunable combinatorial FRET-based bioprobes for cell population analysis of caspase cascades
Source: Sci Rep. 2022 Dec 7;12:21160. doi: 10.1038/s41598-022-25286-z (PMC9729311; doi:10.1038/s41598-022-25286-z)
Supplement: Supplementary file 1 — Supplementary Information. [file 41598_2022_25286_MOESM1_ESM.pdf]

SUPPLEMENTAL INFORMATION, FIGURE TITLES AND LEGENDS

Figure S1. Bioprobe manufacturing processes: (I) Molecule sensor design based on monitoring target and fluorescence coordination: FP was selected on the point of device specifications and molecular sensor coordination. Insertion sequences for recognition of a target and sites into selected FP were narrowed down by referring to previous works and theoretical concerns. Background colors represent basic FP colors as green, yellow and red fluorescent protein. Character colors stand for as follows: black letters denote FP originated amino acid sequences. Blue letters describe spacer amino acid sequences inserted between target recognized sequence (in a red color) and cysteine (in a green color) for chemical modification. Pink letters symbolize a histidine tag (His x 6) for FP purification. (II) Mutant FP production: Genetic constructs encoding mutant FP were developed and transformed into adequate competent bacterial cells. (III) Mutant FP purification: Purified mutant FP was subjected to fluorescence property analysis to estimate misfolded FP content and acquired as qualified fractions. (IV) Selection of dyes for modification: Fluorescence organic dyes to modify purified FP were determined to prepare bioprobes exhibiting high FRET efficiency. (V) Sensing activity check of bioprobes *in vitro*: Sensing performances of bioprobes were investigated to measure FRET efficiency changes upon target enzyme treatments. (VI) feedback reflection: In

case of insufficient sensitivity, selection steps shown with check marks were reconsidered.

Figure S1

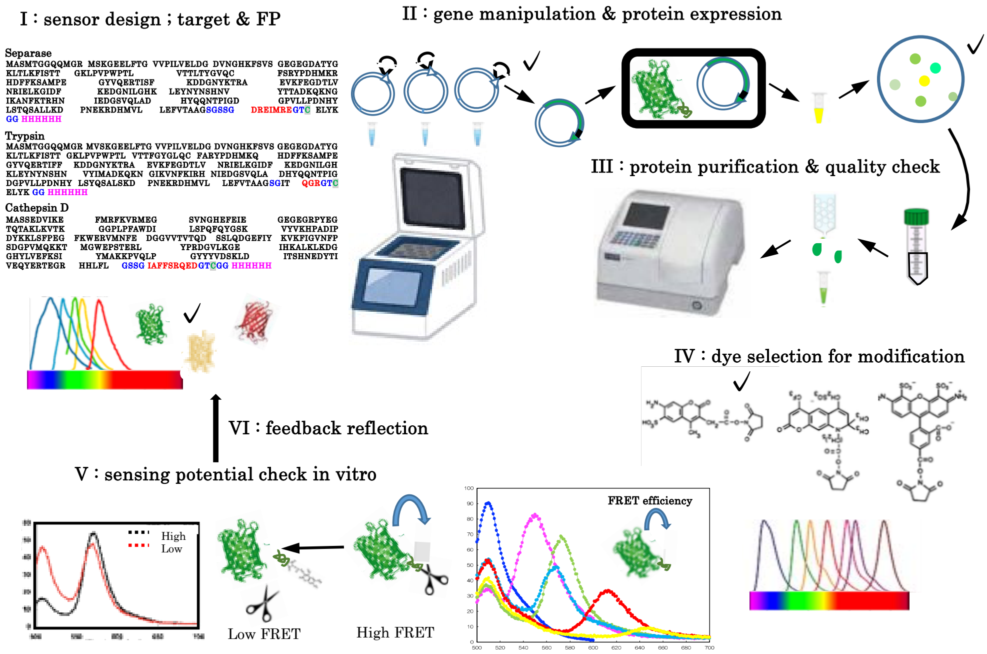

Figure S2. Validation of cell type independent bioprobe performances. Dot plot data through flow cytometric analysis with human epidermal keratinocyte (FEPE1L-8) in A, B, C and mouse embryonic fibroblast (NIH3T3) in D were shown in cases (red; cell population without any bioprobe introduction, sky blue: cell population introduced bioprobes consisting UV5cas12-1 modified with Alexa Fluor 546 (A), UV5casM-22 modified with Alexa Fluor 555 (B), T3castag modified with DyLight Fluor 800 (C) and UV5casS22tag modified with Alexa Fluor 546 (D). Individual sensing targets were indicated at the top of figures. orange: cell population introduced bioprobes and induced apoptosis using same conditions described in materials and methods section)

Figure S2

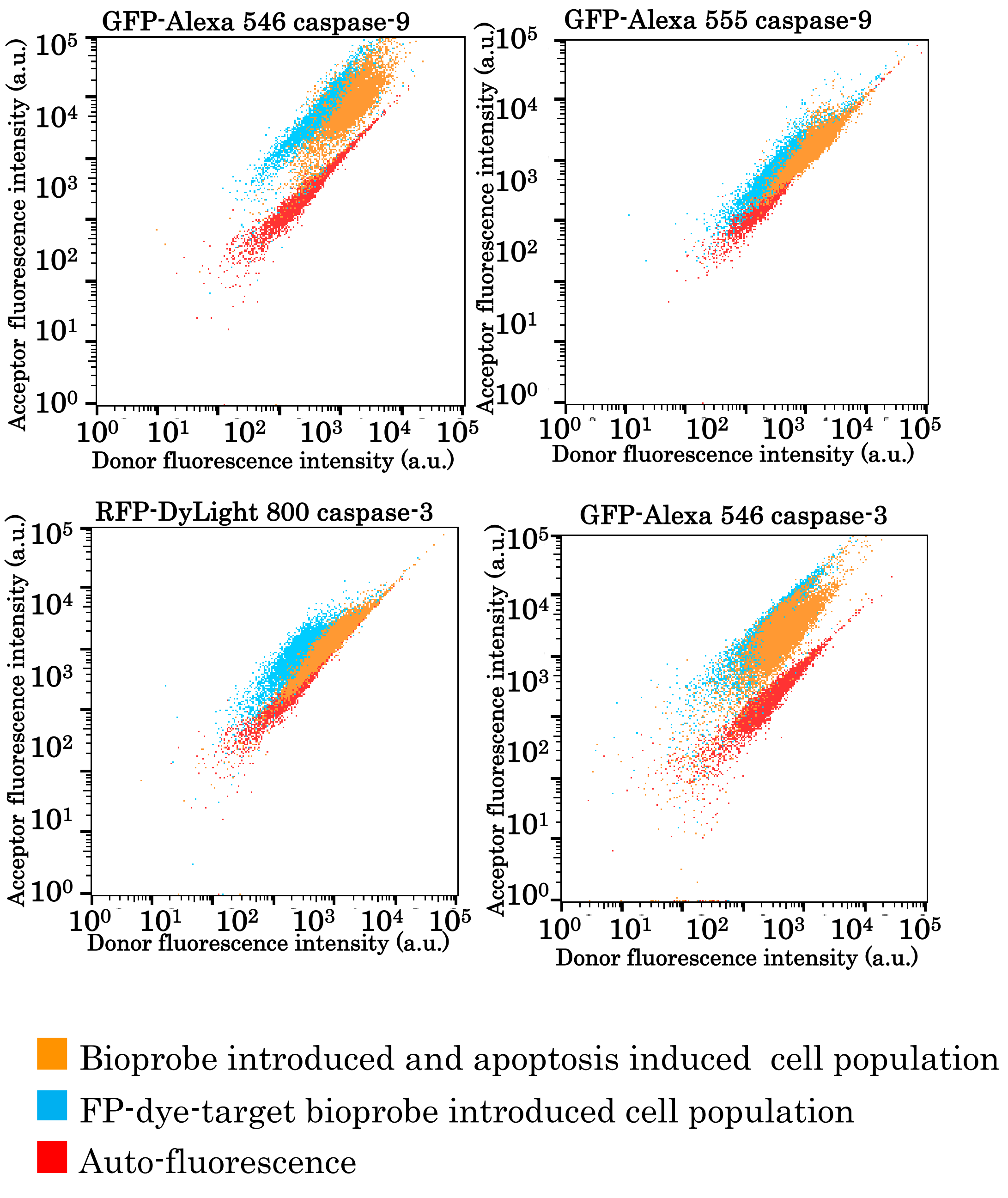

GFP-Alexa 546 caspase-9
GFP-Alexa 555 caspase-9
RFP-DyLight 800 caspase-3
GFP-Alexa 546 caspase-3
Acceptor fluorescence intensity (a.u.)
Donor fluorescence intensity (a.u.)
Bioprobe introduced and apoptosis induced cell population
FP-dye-target bioprobe introduced cell population
Auto-fluorescence

Figure S3. Bioprobe tuning for dual event monitoring and accompanying adjustments of imaging system: (A) Comparison of amino acid sequences of the donor GFP of bioprobe for caspase-9 sensing used in this study (encoded in the pUV5casM-22 plasmid; upper) with those of the one used in the previous study for fluorescence lifetime measurements (encoded in pUV5cas12-1 plasmid; lower). Amino acids sequences of employed basic GFP variants were shown in black and the altered amino acids sequences in this study were in red. (B) Absorption (dotted line) and emission spectrum (solid line) of candidate fluorophores, and laser (440 nm) and filters (ch1-4) used with the microscope in this study. (C) Averaged (left, n=4 cells) and individual (right) response patterns of the caspase-3-sensitive bioprobe introduced into HeLa cells upon induction of apoptosis. Error bars indicate SEM. (D) Fluorescence images of bioprobes introduced into HeLa cells before (left) and after (right) linear spectral unmixing.

Figure S3

A

Caspase-9 (M22)

MASMTGGQQM GRMSKGEELF TGVVPILVEL DGDVNGHKFS
VSGEGEGDAT YGKLTLKFIC TTGKLPVPWP TLVTTLTYGV
QCFSRYPDHM KRHDFFKSAM PEGYVQERTI SFKDDGNYKT
RAEVKFEGDT LVNRIELKGI DFKEDGNILG HKLEYNYNSH
NVYITADKQK NGIKANFKTR HNIEDGSVQL ADHYQQNTPI
GDGPVLLPDN HYLSTQSALL KDPNEKRDHM VLLEFVTAAG
ITHGMDELYK LEHD TC GG HHHHHH

Caspase-9 (12-1)

MASMTGGQQM GRMSKGEELF TGVVPILVEL DGDVNGHKFS
VSGEGEGDAT YGKLTLKFIS TTGKLPVPWP TLVTTLTYGV
QCFSRYPDHM KRHDFFKSAM PEGYVQERTI SFKDDGNYKT
RAEVKFEGDT LVNRIELKGI DFKEDGNILG HKLEYNYNSH
NVYITADKQK NGIKANFKTR HNIEDGSVQL ADHYQQNTPI
GDGPVLLPDN HYLSTQSALL KDPNEKRDHM VLLEFVTAAG
SGSSGIT LEHD GTC ELYK GG HHHHHH

B

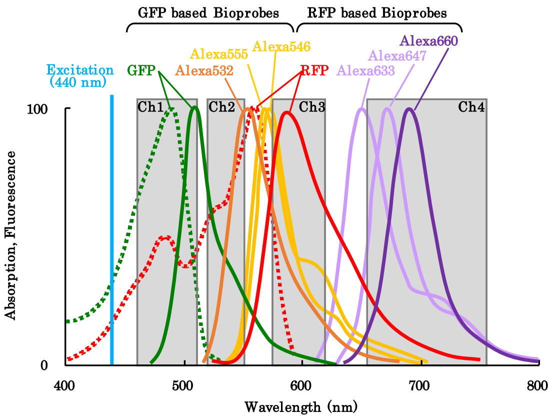

C

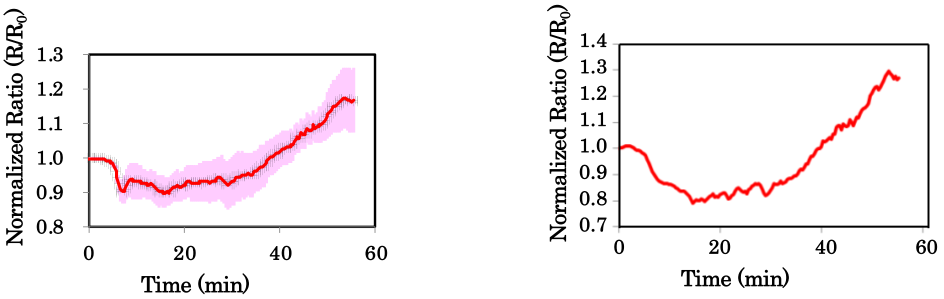

D

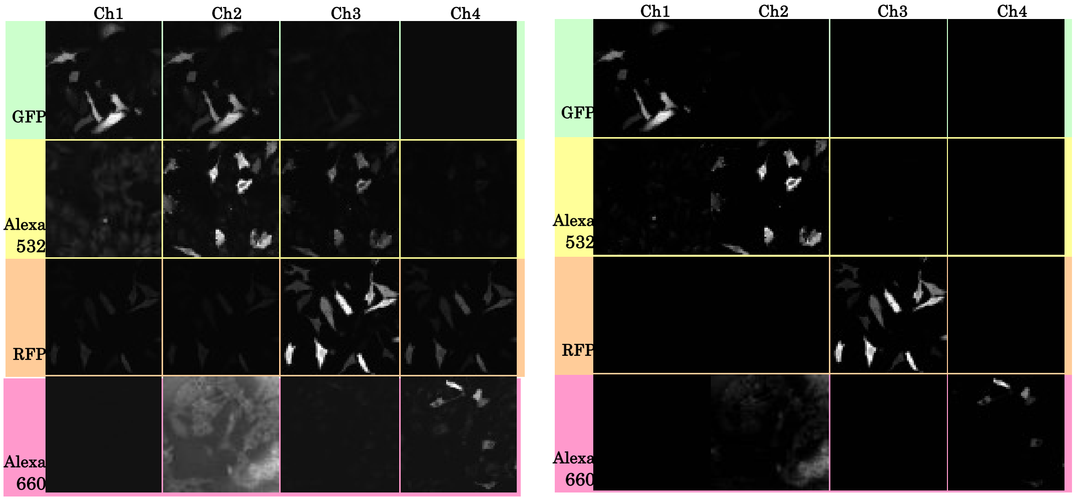

Figure S4. Cellular response heterogeneities: (A-E) ~~Various~~ Five different bioprobe response patterns observed. (A) Pattern I : Caspase-3 activation following caspase-9 activation. (B) Pattern II : Caspase-9 activation only. (C) Pattern III : Capase-3 activation only. (D) Pattern IV : Caspase-9 activation followed by two step caspase-3 activation. (E) Pattern V : No activation. (F) Examples of indicator sets with no correlation. (left) Correlation between response initiation of caspase-3 and response saturation of caspase-9. (middle) Correlation between response saturation of caspase-3 and response. (right) Correlation between response initiation of caspase-3 and response scale of caspase-9.

Figure S4

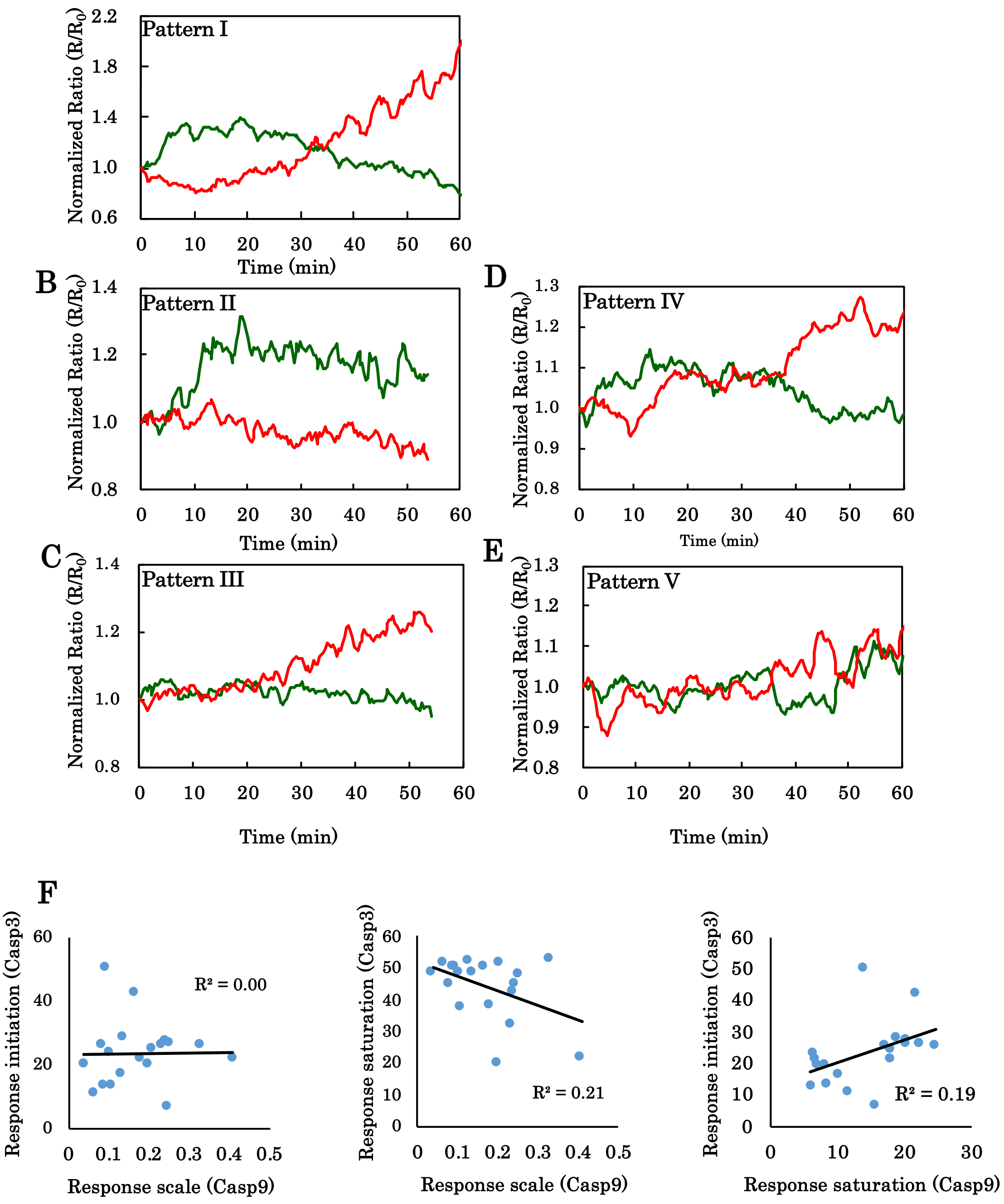
Pattern I
Normalized Ratio (R/R$_0$)
2.2
1.8
1.4
1.0
0.6
0
10
20
30
40
50
60
Time (min)
B
Pattern II
1.4
1.2
1.0
0.8
C
Pattern III
D
Pattern IV
1.3
1.2
1.1
0.9
E
Pattern V
F
Response initiation (Casp3)
Response scale (Casp9)
R² = 0.00
Response saturation (Casp3)
R² = 0.21
Response saturation (Casp9)
R² = 0.19
0.1
0.2
0.3
0.4
0.5

Figure S5. Confirmation of direct bioprobe uptake into HeLa cells through flow cytometry. Dot plot data were shown in cases (gray; cell population without any bioprobe, green: cell population introduced UV5casM-22, orange: cell population introduced UV5casM-22 modified with Alexa Fluor 532, pink: cell population introduced UV5casM-22 modified with Alexa Fluor 555)

Figure S5

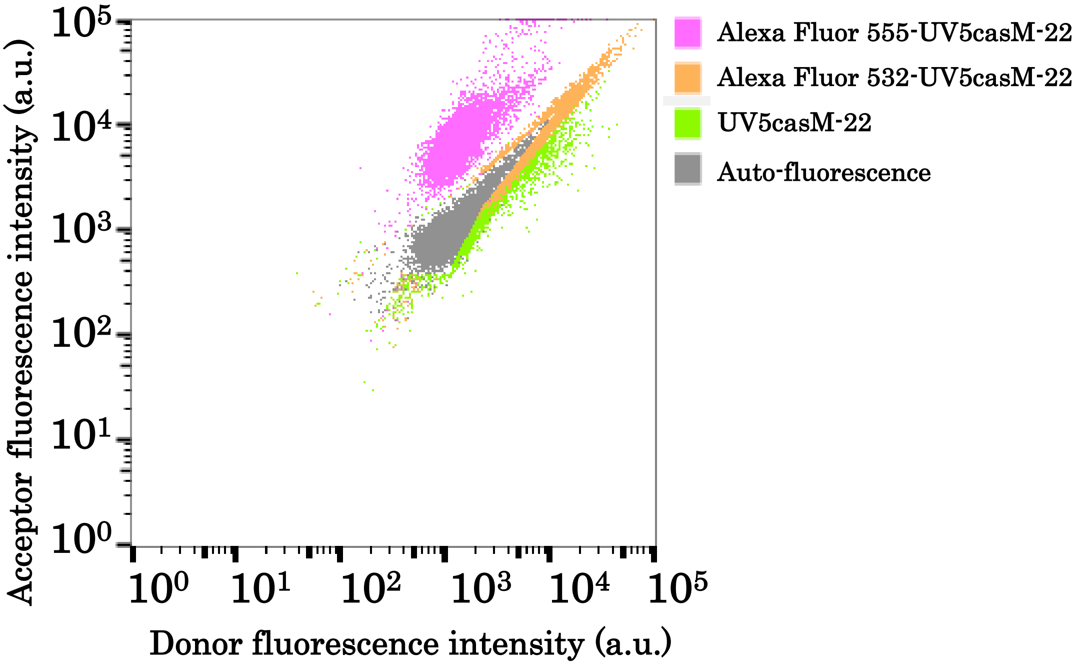

Acceptor fluorescence intensity (a.u.)
10^5
10^4
10^3
10^2
10^1
10^0
10^0 10^1 10^2 10^3 10^4 10^5
Donor fluorescence intensity (a.u.)
Alexa Fluor 555-UV5casM-22
Alexa Fluor 532-UV5casM-22
UV5casM-22
Auto-fluorescence
